# Supplementary material for: Characterization of ORF19.7608 (PPP1), a biofilm-induced gene of Candida albicans
Source: PLoS One. 2025 Nov 11;20(11):e0335473. doi: 10.1371/journal.pone.0335473 (PMC12604798; doi:10.1371/journal.pone.0335473)
Supplement: S2 Fig — (PDF) [file pone.0335473.s002.pdf]

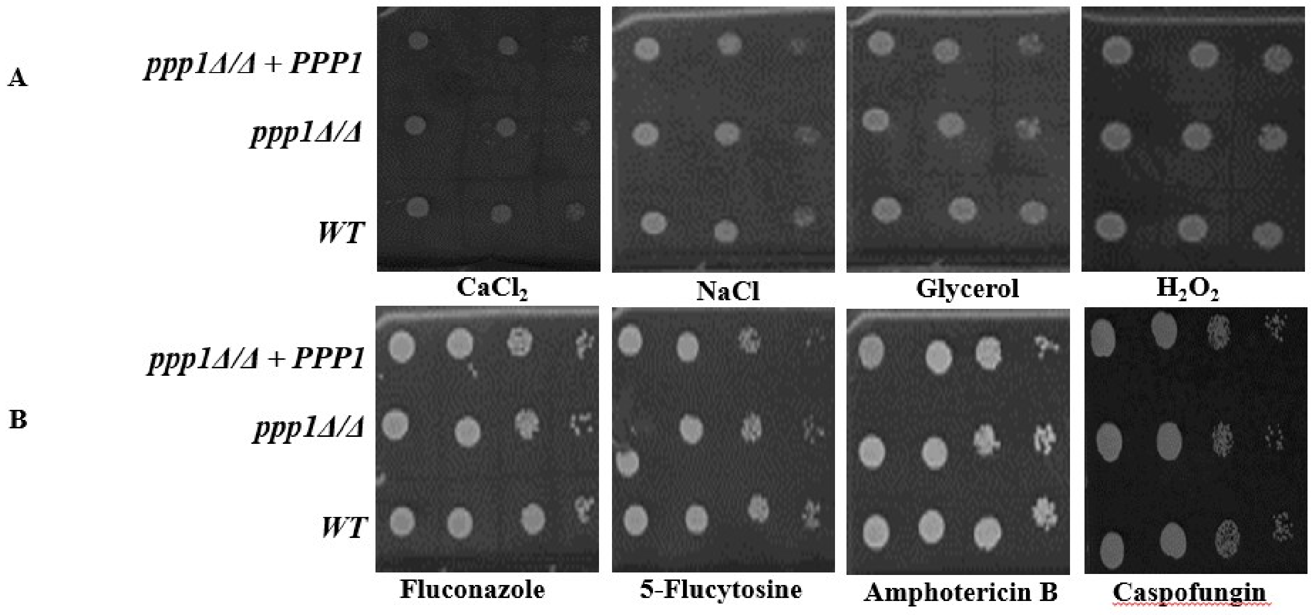

Supplemental Figure S2: Deletion of *PPP1* does not impact stress responses and antifungal resistance

1:10 serial dilution of overnight cultures grown in yeast growth conditions was spotted (highest cell concentrations being 1 x 10^6 cells/ml and lowest being 1 x 10^4 cells/ml or 1 x 10^3 cells/ml) onto YPD agar plates containing different chemicals and incubated at 30°C for 2 days except for caspofungin (5 days).

(A) Strains were subjected to osmotic stress of CaCl2 (400mM), NaCl (500mM), glycerol (100mM) and oxidative stress of H_2O_2 (3mM)

(B) Determination of resistance to different antifungal drugs: fluconazole (1µg/ml), 5-Flucytosine (0.5µg/ml), amphotericin B (0.25µg/ml), and caspofungin (0.75µg/ml).

Experiments were repeated three times for each sample for consistent results. Results were the same at 30^0C and 37^0C
