# Supplementary material for: Characterization of ORF19.7608 (PPP1), a biofilm-induced gene of Candida albicans
Source: PLoS One. 2025 Nov 11;20(11):e0335473. doi: 10.1371/journal.pone.0335473 (PMC12604798; doi:10.1371/journal.pone.0335473)
Supplement: S1 Table — (PDF) [file pone.0335473.s004.pdf]

Supplemental Table 1: Strains Used in this Study

Strains	Parent	Description
ppp1Δ/Δ	*SN148 a/α*	*orf19.7608*Δ::*ARG4*/*orf19.7608*Δ::*ARG4*
ppp1Δ /Δ + *PPP1*	*ppp1Δ/Δ*	*ORF19.7608*/*ORF19.7608*; *his1*/*his1*; leu2/leu2; *arg4/arg4*; *ura3*::imm434/*ura3*::imm434; *iro1*::imm434/*iro1*::imm434 + pV1093-gRNA *ARG4*
PPP1-GFP	*SN148a/α*	*ORF19.7608-GFP-*::*HIS1*/*ORF19.7608-GFP::HIS1*; *leu2/leu2; arg4/arg4; ura3*::imm434/*ura3*::imm434;*iro1*::imm434/*iro1*::imm434
SUR7-Scarlet PPP1-GFP	*PPP1-GFP*	*ORF19.7608-GFP-::HIS1/ORF19.7608-GFP::HIS1*; *SUR7-Scarlet::URA3/ SUR7-Scarlet::URA3; arg4/arg4, leu2/leu2*
PBR1-Scarlet PPP1-GFP	*PPP1-GFP*	*ORF19.7608-GFP-::HIS1/ORF19.7608-GFP::HIS1; PBR1-Scarlet::URA3/ PBR1-Scarlet::URA3; arg4/arg4, leu2/leu2*
ORF19.4654-Scarlet	*SN148a/α*	*ORF19.4654-Scarlet::URA3/ ORF19.4654-Scarlet::URA3; arg4/arg4, leu2/leu2; his1/his1*
