# Supplementary material for: Characterization of ORF19.7608 (PPP1), a biofilm-induced gene of Candida albicans
Source: PLoS One. 2025 Nov 11;20(11):e0335473. doi: 10.1371/journal.pone.0335473 (PMC12604798; doi:10.1371/journal.pone.0335473)
Supplement: S2 Table — (PDF) [file pone.0335473.s005.pdf]

Supplemental Table 2: Oligonucleotides Used in the Study

**Name**	**Sequence (5' to 3')**
***ppp1Δ/Δ*_sgRNA_F**	GATGACGGTGATGATGATGAgttttagagctagaaatagcaagttaaa
***ppp1Δ/Δ*_sgRNA_R**	TCATCATCATCACCGTCATCcaaattaaaaatagtttacgcaagtc
***ppp1Δ/Δ*_repair_F**	TCATTAGCAAAGCAGTTGTAACAAACAAACAAAAAGACAACAATAATAAAAATCCATATCACACAATTACAATAAATCgaagcttcgtacgctgcaggtc
***ppp1Δ/Δ*_repair_R**	TGAAATAAAACAAAAATAAAACAACACAAAGTTGAATAAGAAGAAGAAAGATTCAAATAGTACGGCTTGGCTATGTtctgatatcatcgatgaattcgag
***ppp1Δ/Δ*_diag_F**	GGAGTCGTCAACTGCAAATTGTGAAG
***ppp1Δ/Δ*_diag_F**	ATTAGCCAAGGCAGGATGTATAGC
***ppp1Δ/Δ+PPP1*-FW**	gtcgacTGACTGTTTGTTTCAATTC
***ppp1Δ/Δ+PPP1*-Rv**	acgcgtGCAGATAGTGGCATG
**Fw_Int_*ppp1Δ/Δ+PPP1***	GAAGACTTCAGTCGTTTTAGCTGC
**RPF1-R**	CGCCAAAGAGTTTCCCCTATTATC
**RPF-1**	GAGCAGTGTACACACACACATCTTG
**Diag_RP10_F**	CATGAGGCCTCCATGAGGCCTC
**Diag_CIp10_R**	agctatgaccatgattacgccaagc
***URA3*-F**	ggagttggattagatgataaaggtgatgg
***PPP1-GFP*_sgRNA_F**	GTAGTGAATATATTTGAAAAgttttagagctagaaatagcaagttaaa
***PPP1-GFP*_sgRNA_R**	TTTTCAAATATATTCACTACcaaattaaaaatagtttacgcaagtc

***PPP1-GFP*_repair_F**	GAGATTACTTTTCTCAATGGAAACAAGGACTCGACAACTTAATTCAA AAAGGTAAGACATGGTTTAGTGGTCTTTTCGGTggtgctggcgcaggtgcttc
***PPP1-GFP*_repair_R**	ATATTATGAGAAAAAAAATTTGATATACCTAAGAATTGAAACCTGTAA ATGACAAAAAATTTATAAACAAATAAAAATCCccgcataggccactagtgga
***PPP1-GFP*_Diag_Int_F**	GAAGACTTCAGTCGTTTTAGCTGC
**Con_*PPP1*-R1**	ATTAGCCAAGGCAGGATGTATAGC
***PPP1-GFP*_Diag_Ext_F**	TGAAGCACCTTCTTTACAGCAACACG
***GFP*_R**	tctttcgaaagggcagattgtgtgg
***HIS1*_F**	gcagatggcgagtacgaaaagc
***PPP1-GFP*_Diag_Ext_R**	CAACTTTCACAAGTGCAGATAGTGGCA
***SUR7*-Scarlet_sgRNA_F**	CGTATATTAAATATACCAATgttttagagctagaaatagcaagttaaa
***SUR7-Scarlet*_sgRNA_R**	ATTGGTATATTTAATATACGcaaattaaaaatagtttacgcaagtc
***SUR7-Scarlet*_repair_F**	CAGGCGGTATTAGATTCTTCAAAATCAAAAGAAACCAAAAAGTTTCC GATGATGAATCAGTAggtgctggcgcaggtgctatggtcagtaaaggggaagc
***SUR7*-Scarlet_repair_R**	TAAAGATTCCAATAATGGTAATACTGATAATAATAATAATAATAACAAT GATGATTTTAGTAATAGTAGTAACAGTtctgatatcatcgatgaattcgag
***SUR7-Scarle*t_diag_ext_F**	GTCTCATTGCCCTTGCATTCAGTG
***SUR7-Scarlet*_diag_ext_R**	CACAATCCATATGTAACTCATGTCACG
**Diag_*Scarlet*_R2**	GGCATTTGCACAGGTTTCTTAGC
**Diag_*URA3*-F1**	gaaactcatgcctcaccagtagc

***ORF19.4654-Scarlet*_sgRNA1_F**	CTTCTCTGTACATTAAGTTAgttttagagctagaaatagcaagttaaa
***ORF19.4654-Scarlet*_sgRNA1_R**	TAACTTAATGTACAGAGAAGcaaattaaaaatagtttacgcaagtc
***ORF19.4654-Scarlet*_sgRNA2_F**	CTACTCATTGGTTGTTCTAGgttttagagctagaaatagcaagttaaa
***ORF19.4654-Scarlet*_sgRNA2_R**	CTAGAACAACCAATGAGTAGcaaattaaaaatagtttacgcaagtc
***ORF19.4654-Scarlet*_repair_F**	ATATTCAATGGAAATTATTATTAGGTTGTTTTTATTTGCAATTGTAAGT TTACTTGCAATGggtgctggcgcaggtgctatggtcagtaaaggggaagc
***ORF19.4654-Scarlet*_repair_R**	TAGAACTGAATCTGGGTATATTTTTTGTTAATTTCGGTCCGATTTAGCT ATTTGCTATGGTATTAAGTTCCCCATTtctgatatcatcgatgaattcgag
***ORF19.4654-Scarlet*_diag_ext_F**	CAGCAACAACATTGTCAACCACCAA
***ORF19.4654-Scarlet*_diag_ext_R**	CGCGCGTTCTTTTCTCCTCCT
***PBR1-Scarlet*_sgRNA_F**	GTTTAATTAATTGTTAAGTTgttttagagctagaaatagcaagttaaa
***PBR1-Scarlet*_sgRNA_R**	AACTTAACAATTAATTAAACcaaattaaaaatagtttacgcaagtc
***PBR1-Scarlet*_repair_F**	ATCTGCCACCAAAGAACTCCTACAAAGTTAGTATCTACGGTCGTCTCA ATTGGGCTGTCTTGggtgctggcgcaggtgctatggtcagtaaaggggaagc
***PBR1-Scarlet*_repair_R**	TTATATATATATGTACACATAGTATAAGAACTAATAAATAAGCAACGAG AAGAAACGAAATAAAACAACGACAACGtctgatatcatcgatgaattcgag
***PBR1-Scarlet*_diag_ext_F**	GATCAAAAGAGTGGTGAAATCAAA
***PBR1-Scarlet*_diag_ext_R**	GGGCTCAAGGGTAACTCTTCTTT

Capital letters represent genomic sequence

Small letters represent plasmid sequences, a linker, or a restriction site added

sgRNA_R: P2 [11]

sgRNA_F: P3 [11]

Plasmids

pV1093

CIP10-mScarlet-IDT

pFA-ARG4

pFA-GFP-CAURA3

pFA-GFP-HIS1
